# Associations Between Sexualized Media Consumption, Sexual Double Standards, and Sexual Coercion Perpetration and Victimization in Late Adolescent Sexually Active Boys and Girls from The Netherlands

**DOI:** 10.1007/s10508-024-02988-1

**Published:** 2024-09-03

**Authors:** Faye Chadwick-Brown, Joyce J. Endendijk

**Affiliations:** Child and Adolescent Studies, Faculty of Social and Behavioral Sciences, Utrecht University, Heidelberglaan 1, P.O. Box 80140, 3508 TC Utrecht, The Netherlands

**Keywords:** Adolescence, Sexual coercion, Sexualized media, Sexual double standards

## Abstract

This study examined associations between sexualized media consumption, sexual double standard (SDS) norms, and sexual coercion perpetration and victimization in late adolescence and whether these associations were moderated by gender. Participants were sexually active Dutch secondary school students aged 16–20 years (*N* = 255, 58.4% girls). Data were collected using a self-report questionnaire at a single time-point. Overall, more sexualized media consumption was associated with higher odds of both sexual coercion perpetration and victimization. These associations did not differ by gender. Specifically, viewing online pornography and sexually oriented reality television more frequently were associated with higher odds of sexual coercion perpetration, whereas viewing others’ sexy social media posts more frequently was associated with higher odds of sexual coercion victimization. Finally, stronger endorsement of SDS norms was associated with lower odds of sexual coercion perpetration in girls, but with comparatively higher odds of sexual coercion perpetration in boys. Results indicate that consuming sexualized media and societal sexual double standards is associated with an increased risk of sexual coercion experiences in adolescence.

## Introduction

Sexual coercion involves pressuring someone into unwanted sexual activity, for example through lies or false promises, threats to end a relationship, consistent verbal pressure, or use of drugs or alcohol (Smith et al., 2017; Young et al., 2012). Understanding correlates of sexual coercion is vital, especially in adolescence which represents a period of major sexual developments, thereby providing a window of opportunity for intervention efforts to decrease rates of sexual coercion perpetration and victimization (Collins et al., 2009; De Graaf et al., 2017; DeLamater & Friedrich, 2002). Sexual coercion affects large numbers of adolescents cross-culturally. For instance, among Spanish adolescents, 48% of boys and 28% of girls reported perpetrating sexual coercion when their sexual advances were rejected, whilst 49% of boys and 47% of girls who rejected others’ sexual advances reported being victimized (Fernández-Fuertes et al., 2018). In the United States, 41% of adolescents were sexual coercion victims by the end of high school (Young et al., 2012). Victimization is often followed by increases in externalizing symptoms and sexual risk behaviors, such as a higher number of sexual partners and greater intercourse frequency (Young et al., 2012).

In this study, associations were examined between sexualized media consumption (online pornography, sexually oriented reality television, sexually explicit music videos, and others’ sexy social media posts), stereotypes about the sexual double standard, and both sexual coercion perpetration and victimization. Several media types were selected due to their popularity among adolescents. In the Netherlands, adolescents use a screen for 6–7 h per day on weekdays and 5.4 h per day on weekends (van Rooij et al., 2020). Time spent on social media is increasing, with TikTok and Snapchat recently gaining popularity among Dutch adolescents (Newcom, 2023). In the US, adolescents spend almost 1 h per day watching online videos, almost 2 h watching television, over 1 h using social media, and over 2 h listening to music (Rideout & Robb, 2019). In addition, 73% of US adolescents aged 13–17 years have viewed online pornography (Robb & Mann, 2023).

Whilst reality television, sexualized music videos, and pornography have previously been studied in relation to sexual coercion, viewing others’ sexualized social media posts has not explicitly been linked to perpetration or victimization. In addition, most studies examined one media type, one gender, or studied either perpetration or victimization. Typically, studies examine male perpetrators or female victims, despite female perpetration and male victimization being common (Fernández-Fuertes et al., 2018). This study therefore advances the literature by asking both boys and girls about both perpetration and victimization, and by including multiple types of sexualized media. It is important to understand which media types are most relevant, so that intervention programs aimed at reducing sexual coercion among adolescents know where to intervene.

### Sexualized Media and Sexual Coercion: Theoretical Foundations

There are two main theories that may explain the associations between sexualized media consumption and sexual coercion perpetration and victimization: social learning theory (Bandura, 1977) and objectification theory (Fredrickson & Roberts, 1997). Social learning theory proposes modeling, a process of observing and learning from others’ behaviors and their consequences within a social context, as a central mechanism through which individuals learn to perform behaviors (Bandura, 1976). Modeling of sexual coercion should be more likely when the media explicitly depicts sexually coercive interactions (Bandura, 1965). Online pornography explicitly depicts coercion, exploitation, and violence (Vera-Gray et al., 2021), with 88% of popular pornographic films containing physical aggression and 49% containing verbal aggression, typically by a man towards a woman (Bridges et al., 2010). Viewers may model coercive behaviors, particularly when they are rewarded (Bandura, 1965). This reward is provided in pornography through targets of sexual aggression responding by expressing pleasure (Bridges et al., 2010). Of adolescents who reported viewing violent pornography, 61% of boys and 44% of girls thought that the women appeared to enjoy the violence inflicted upon them (Romito & Beltramini, 2015). Sexually oriented reality television has been criticized in the media for normalizing sexually coercive behaviors (Birdsall & Keay, 2018; Morgan, 2021). Although academic research on this topic is lacking, sexually oriented reality television has the potential to depict sexually coercive interactions as it centers on the sexual or romantic relationships of the participants.

Objectification theory (Fredrickson & Roberts, 1997) may also explain associations between several types of sexualized media consumption and sexual coercion. Objectification theory focuses on the sociocultural context in which the physical body is placed. It proposes that, in a culture dominated by heterosexuality, female bodies are constantly looked at and evaluated, resulting in the potential for sexual objectification (Fredrickson & Roberts, 1997). Sexual objectification involves a focus on physical beauty and sexual appeal that reduces subjects to an object for others’ sexual use and particularly affects women (American Psychological Association, Task Force on the Sexualization of Girls, 2007; Galdi & Guizzo, 2021). Sexual objectification denies the objectified individual personhood (Loughnan et al., 2010) and agency (Cikara et al., 2011). It may therefore contribute to increased sexual coercion perpetration risk through altering the perpetrator’s perception of the victim’s right and ability to (not) consent to sexual activity. Previous research has established links between consuming objectifying media, objectification, and attitudes supportive of sexual violence (Wright & Tokunaga, 2016), as well as between objectification and sexual coercion perpetration (Sáez et al., 2019). Objectification is prevalent in several types of media. For instance, in online pornography, women are used for men’s pleasure and are objectified through frequent close-ups of their sexual body parts (Klaassen & Peter, 2015). Music videos in the R&B and hip-hop genres often depict women as primarily decorative (Stevens Aubrey & Frisby, 2011). On US primetime television, male characters judge women by their physical appearance and treat them as sexual objects (Kim et al., 2007). In Instagram “fitspiration posts”, 48% of women and 29% of men are sexualized (Carrotte et al., 2017).

Not surprisingly, consuming sexualized media is associated with greater self-objectification, that is the internalization of objectification (Fredrickson & Roberts, 1997), for both men and women (Karsay et al., 2018). Adolescents who have internalized sexual objectification are more likely to present themselves in a more sexualized way (Liss et al., 2011; Visser et al., 2022). This may in turn increase their risk of being objectified by others (Cikara et al., 2011), which could increase victimization risk. Self-objectification is also associated with stronger approval motivation (Chen et al., 2022), which could lead adolescents to prioritize their partner’s feelings over their own and possibly increase the risk of becoming a victim of sexual coercion. Self-objectification is common in the media types examined in the current study. Female music artists (Stevens Aubrey & Frisby, 2011) and female television characters (Kim et al., 2007) frequently self-objectify. In a content analysis of reality television programs, 47% of women wore tight, revealing outfits, compared to 2% of men (Bergstrom, 2005), although an analysis of “Jersey Shore” found that men also wore highly sexualized clothing (Anderson & Ferris, 2016). Moreover, of Instagram images posted by young women, 30% were self-objectifying (Bell et al., 2018).

Therefore, as women are more often explicitly victimized by male perpetrators, as well as (self-)objectified more than men in the media, we hypothesized that associations between sexualized media consumption and sexual coercion perpetration would be stronger for adolescent boys than girls, and that associations between sexualized media consumption and sexual coercion victimization would be stronger for adolescent girls than boys.

### Sexualized Media and Sexual Coercion: Previous Findings

Previous research identified significant associations between sexualized media consumption and either sexual coercion perpetration or associated attitudes. Unfortunately, many studies included only male participants. Specifically, watching sexualized music videos was associated with greater acceptance of rape myths (Kistler & Lee, 2009) and greater acceptance of violence against women (Stevens Aubrey et al., 2011) among male emerging adults. Reality television consumption was also associated with greater acceptance of rape myths and more frequent sexual coercion perpetration in male emerging adults (Seabrook et al., 2019). Pornography consumption was associated with increased sexual coercion perpetration in European adolescent boys (Stanley et al., 2018) and US male (Simons et al., 2012) and female (Kernsmith & Kernsmith, 2009) emerging adults. Effects among adolescent girls were less clear as few reported regular pornography consumption (Stanley et al., 2018).

Previous research also identified significant associations between sexualized media consumption and either sexual coercion victimization, other types of sexual victimization or associated attitudes. Specifically, greater consumption of sexualized music videos was associated with increased odds of rape victimization (Ybarra et al., 2014). In addition, greater consumption of sexualized television and film was associated with increased odds of sexual coercion victimization (Ybarra et al., 2014). Moreover, for women, sexual coercion (Simons et al., 2012) and sexual assault (de Heer et al., 2021) victimization were more likely among those who consumed pornography. Similarly, among adolescent boys, a study of violent pornography consumption found that it was associated with increased odds of sexual dating violence victimization (Rostad et al., 2019). Furthermore, consuming reality television predicted acceptance of sexualized aggression in female emerging adults (Papp et al., 2022), but the reverse path was not significant, which provides evidence for the directionality of the association. It is unclear how reality television consumption relates to sexual coercion victimization in men. Thus, the existing evidence points towards sexualized media consumption being associated with greater likelihood of sexual violence perpetration and victimization in both men and women. Yet, the limited attention to, and inconclusiveness of, gender differences in this body of research warrants further scrutinizing of gender as a moderator in the associations between exposure to sexualized media and both sexual coercion perpetration and victimization.

### Sexual Double Standard and Sexual Coercion

Another important factor in adolescents’ sexual coercion perpetration and victimization is the sexual double standard (SDS). The sexual double standard can be considered a social norm that dictates different sexual behaviors for men and women (Emmerink et al., 2017; Endendijk et al., 2020, 2022). According to the SDS, men/boys are expected to be highly sexually active, dominant, and the initiator of (hetero)sexual activity, whereas women/girls are expected to be sexually reactive, submissive, and passive. Men are also granted more sexual freedom than women. Once internalized in sexual scripts or schemas, such social norms provide guidelines for men’s and women’s expectations and behaviors in sexual situations (Boahene et al., 2022). Because traditional SDS norms and scripts dictate passivity for females and dominance by males in sexual interactions, women might be particularly vulnerable to sexual victimization by male perpetrators (Krahé et al., 2000). Indeed, there are clear gender differences in the perpetration and victimization of sexual violence, with about 90% of victims of rape being female (U.S. Department of Justice, 2013). Similarly, 11 to 16% of adolescent and emerging adult boys report perpetration of sexual coercion, compared to four to five percent of girls (Krahé et al., 2015; Shen et al., 2012). Gender differences in victimization are often smaller, with reported frequencies ranging from 11 to 27% for adolescent and emerging adult males and from 9 to 32% for females (Krahé et al., 2015; Shen et al., 2012).

To the best of our knowledge, no research has directly studied whether the association between adolescents’ endorsement of SDS norms and sexual coercion perpetration and victimization are different for boys and girls. As boys and girls who endorse traditional SDS norms expect males to be sexually dominant and females to be sexually passive, we suspect that SDS norms may be more strongly associated with sexual coercion perpetration in boys and with sexual coercion victimization in girls. There is some evidence that traditional gender norms have been associated with increased risk for sexual coercion perpetration in adolescent boys but not in girls (Shen et al., 2012). Similarly, less gender equality in a country, which is associated with a more pronounced SDS (Endendijk et al., 2020), was related to higher rates of sexual aggression perpetration by males compared to females, but also to higher male victimization rates (Krahé et al., 2015). Finally, hostile sexist attitudes were linked to sexual coercion perpetration by adolescent boys and girls (Fernández-Fuertes et al., 2018). Thus, previous research seems to be inconsistent as to whether gender norms and attitudes might be associated with sexual coercion perpetration and victimization in different ways for men and women. Studying the specific links between adolescents’ endorsement of the SDS and the sexual coercion perpetration and victimization of boys and girls might provide clarity in this regard.

### Interaction Between Sexualized Media and Sexual Double Standard

It is unclear how associations between sexualized media consumption and sexual coercion may be moderated by individual beliefs, as potential moderators have received little attention (Rodenhizer & Edwards, 2019). However, media effects are theorized to be more likely when media content and personal beliefs are congruous, partly due to faster and more efficient cognitive processing (Valkenburg & Peter, 2013). The media often depicts a world where men should be sexually assertive and women are viewed as passive sexual objects, aligning with the traditional SDS norms of men as sexually active and dominant and women as reactive and passive (Kim et al., 2007; Ward, 1995). More specifically, girls are more likely than boys to present themselves in a sexy way on social media (Vandenbosch et al., 2015) and men are depicted as sex-driven whereas women are depicted as sexual objects in pornography (Klaassen & Peter, 2015), many music videos, and fiction TV genres (Ward, 2003). This also seems to be true of reality television. An analysis of “Love Island UK” found that men were presented as occupying a dominant role in sex and celebrated for their sexual prowess, whilst women were shamed and labelled “man-eaters” if they were sexually dominant (Denby, 2021). Therefore, in line with the differential susceptibility to media effects model (Valkenburg & Peter, 2013), we expected that associations between sexualized media consumption and sexual coercion would be stronger when adolescents more strongly endorse traditional SDS norms.

### Current Study

This study examined associations between sexualized media consumption, SDS norms, and sexually active adolescents’ self-reported sexual coercion perpetration and victimization. We also explored whether these associations were moderated by adolescent gender and whether there was an interaction between sexualized media consumption and SDS norms.

The following hypotheses were tested:

1. As consumption of sexualized media increases, adolescents will have greater odds of sexual coercion perpetration and sexual coercion victimization. This would especially be the case for adolescents who more strongly endorse SDS norms.
2. Associations between sexualized media consumption and sexual coercion perpetration would be stronger for boys than girls.
3. Associations between sexualized media consumption and sexual coercion victimization would be stronger for girls than boys.
4. Associations between endorsement of SDS norms and sexual coercion perpetration would be stronger for boys than girls.
5. Associations between endorsement of SDS norms and sexual coercion victimization would be stronger for girls than boys.

## Method

### Participants

Classes from 24 Dutch high schools and lower vocational schools (in Dutch: MBO) were recruited by student assistants from Utrecht University’s Clinical Child, Family, and Education Studies, through their personal networks via convenience sampling. Schools received information letters in-person or through e-mail. Twenty-two schools had one participating class and two schools had two participating classes. Data were collected between November 2017 and June 2019.

In total, 699 adolescents participated. Those younger than 16 years and older than 20 years were excluded for being outside the target age range. Of the remaining 611 participants, 255 reported sexual experiences with another person (e.g., touching while naked, oral sex, penetrative sex). Sexual experience was a prerequisite for inclusion, as we were interested in understanding factors that contribute to different sexual coercion perpetration and victimization experiences among adolescents who have been in situations where sexual coercion is likely to occur. These 255 participants completed all study measures and comprise the final sample. There was no missing data. Table 1 presents their background characteristics. Boys and girls did not differ in age (*p* = .451), educational level (*p* = .242), or ethnicity (*p* = .098).

**Table 1:** Demographic characteristics of participants

Sample characteristics	Total sample	Girls	Boys
*n* (%)	255 (100)	149 (58.4)	106 (41.6)
Age (in years), *M* (*SD*)	17.37 (1.02)	17.41 (1.05)	17.31 (0.98)
Educational level, *n* (%)			
Lower secondary or vocational education	118 (46.3)	72 (48.3)	46 (43.4)
Higher secondary education	87 (34.1)	54 (36.2)	33 (31.1)
Pre-university education	47 (18.4)	22 (14.8)	25 (23.6)
Gymnasium/Grammar school	3 (1.2)	1 (0.7)	2 (1.9)
Ethnicity, *n* (%)			
Dutch	199 (78.0)	117 (78.5)	82 (77.4)
Moroccan	0 (0.0)	0 (0.0)	0 (0.0)
Turkish	4 (1.6)	3 (2.0)	1 (0.9)
Surinamese	14 (5.5)	8 (5.4)	6 (5.7)
Asian	1 (0.4)	0 (0.0)	1 (0.9)
Indonesian	12 (4.7)	3 (2.0)	9 (8.5)
Other	25 (9.8)	18 (12.1)	7 (6.6)
Sexual coercion			
Reported perpetration, *n* (%)	38 (14.9)	16 (10.7)	22 (20.8)
Reported victimization, *M* (*SD*)	0.45 (0.67)	0.57 (0.72)	0.27 (0.56)

For the Reported victimization measure, absolute range is 0–2

### Procedure

Participants completed an online questionnaire through Limesurvey. This took approximately 45 min and was completed during class time, under the supervision of a student assistant. Questions were in the same order for all participants. Questions about media consumption were presented after questions about the adolescents’ endorsement of SDS norms, so that media stereotyping did not unduly influence participants’ reporting of their own views about the SDS. Sensitive questions about sexual experience and sexual coercion were placed towards the end of the questionnaire. Before the sexual experience and coercion questions, it was explained that “sex” meant everything ranging from naked touching and caressing to intercourse. All questions were either from previously validated questionnaires or were adaptations of validated questionnaires. Adolescents were not compensated for their participation.

### Measures

#### Sexual Coercion

Adolescents’ experiences with sexual coercion were assessed with three questions used in previous research (Collibee & Furman, 2014; Eaton & Matamala, 2014). Sexual coercion perpetration was measured using one question (Have you ever applied some pressure to get someone to have sex with you?). Sexual coercion victimization was measured using two questions (Have you ever felt forced to have sex when you didn’t really want to?, Have you ever had sex against your will while under the influence of alcohol or drugs?). Participants responded yes (1) or no (0) to all three questions. Scores for the two victimization questionnaires were summed, resulting in a score range from 0 to 2. The perpetration variable was dichotomous.

#### Exposure to Sexually Explicit Music Videos

Adolescents’ self-reported how regularly, in the past 6 months, they had watched music videos by three male artists (e.g., Drake, Justin Bieber, Ronnie Flex) and three female artists (e.g., Rihanna, Ariana Grande, Nicki Minaj) (van Oosten et al., 2015a, 2015b). Artists were selected using three criteria: (1) they released R&B, hip-hop, or rap music (genres known for sexual content, objectifying content, and hypermasculine/hyperfeminine portrayals of men and women) (Avery et al., 2017; Hansen & Hansen, 2000; Smiler et al., 2017; Stevens Aubrey & Frisby, 2011; Turner, 2011; Wright, 2009) and released music videos containing sexual content, (2) they were popular among Dutch adolescents, and (3) they were established in the Dutch music charts. Participants responded on a 7-point scale, ranging from 1 (never) to 7 (several times a day). The six items were averaged to create one variable representing exposure to sexually explicit music videos (Cronbach’s *α* = 0.77).

#### Exposure to Online Pornography

Adolescents’ self-reported how regularly, in the past 6 months, they had intentionally watched Internet content containing pictures and videos with clearly exposed genitals, and pictures and videos in which people had sex (Vandenbosch et al., 2015). Participants responded on a 7-point scale, ranging from 1 (never) to 7 (several times a day). The four items were averaged to create one variable representing exposure to online pornography (Cronbach’s *α* = 0.91).

#### Exposure to Reality Television

Adolescents self-reported how regularly, in the past 6 months, they had watched six reality television shows (e.g., MTV’s Geordie Shore, MTV’s Ex on the Beach) (Vandenbosch et al., 2015). Shows were selected using three criteria: (1) they showed explicit sexual content and alcohol use; (2) they were popular among Dutch adolescents, and (3) they were broadcast before and during data collection. Participants responded on a 7-point scale, ranging from 1 (never) to 7 (every episode). The six items were averaged to create one variable representing exposure to sexually oriented reality television (Cronbach’s *α* = 0.70).

#### Exposure to Sexy Online Presentation of Others

Adolescents self-reported how regularly, in the past 6 months, they had seen photographs on social media sites (e.g., Facebook, Instagram) where girls/women and boys/men presented themselves (1) with a sexy gaze, (2) scantily dressed (e.g., in underwear or swimwear), (3) with a sexy appearance, and (4) in a sexy pose (van Oosten et al., 2015a, 2015b). Participants responded on a 7-point scale, ranging from 1 (never) to 7 (several times a day). The eight items were averaged to create one variable representing exposure to others’ sexy social media posts (Cronbach’s *α* = 0.91).

#### Adolescent Gender

Adolescents self-reported their gender (0 = boy, 1 = girl).

#### Endorsement of Sexual Double Standard

An adapted version of the Emmerink et al. (2017) Scale for the Assessment of the Sexual Standards Among Youth was used (Endendijk et al., 2022). Participants indicated who is more likely to exhibit certain sexual behaviors (e.g., Who do you expect to more often have sex without love?) on a 3-point scale (0 = both genders equally often/neither gender, 1 = boys/men, 2 = girls/women). Items were recoded so that positive scores (+ 1) represent stereotypic expectations, neutral scores (0) represent egalitarian expectations, and negative scores (−1) represent counter-stereotypic expectations. This adapted scale was previously shown to load onto a single factor, except for the item “looking attractive” (Endendijk et al., 2022). As “looking attractive” is the only item that does not measure a sexual behavior, it was removed from our measure. The recoded scores of the remaining 15 items were averaged to create one variable representing adolescents’ endorsement of SDS norms (Cronbach’s *α* = 0.75).

### Statistical Analyses

Data were prepared and analyzed using IBM SPSS Statistics (Version 28). First, several descriptive analyses were used. Associations and potential multicollinearity between the independent variables were examined using Pearson correlation. Gender differences on the study variables were examined using independent sample *t* tests for continuous variables and chi-square tests for categorical variables.

Second, due to sample size limitations and low frequency of sexual coercion perpetration and victimization it was necessary to reduce the number of predictors and interactions in each analysis. Therefore, an overall sexualized media exposure score was created by standardizing and averaging the separate media type variables. This method is common in the literature examining effects of media exposure (Vandenbosch & Eggermont, 2016; Vossen & Fikkers, 2021). When significant associations with this overall sexualized media exposure variable were found, post-hoc analyses were conducted to examine which types of sexualized media were driving the associations. A binary logistic regression analysis was conducted for the binary sexual coercion perpetration variable and an ordinal logistic regression was used for the ordinal victimization variable. In both analyses, participant gender and relevant covariates were added in the first step of the model. In the second step, sexualized media exposure and SDS norms were added. Finally, in the third step, three two-way interactions between gender, sexualized media exposure, and SDS norms were included (i.e., gender * sexualized media exposure, gender * SDS norms, sexualized media consumption * SDS norms). Inclusion of covariates (age, educational level, ethnicity) was determined separately for each analysis based on the change-in-estimate method, > 5% criterion (Rothman et al., 2008).

## Results

### Assumption Checks

No outliers were identified on any variables. Standardized skewness and kurtosis values indicated that consumption of other’s sexy social media posts and endorsement of SDS norms were normally distributed. Consumption of sexually explicit music videos, online pornography, and sexually oriented reality television had positive skew. Binary logistic and ordinal regression analyses do not require variables to be normally distributed. Correlations between the predictors did not indicate any problematic multicollinearity (all correlations < 0.23). For the binary logistic regression analyses, interactions of predictors with their natural logs were non-significant (*p*-level adjusted to 0.004, Tabachnick & Fidell, 2014). The assumption of linearity in the logit was therefore met. For the ordinal regression analyses, tests of parallel lines were non-significant. The assumption of proportional odds was therefore met.

As individual adolescents were nested in schools, it was checked whether multilevel modeling was necessary to take into account possible dependency in the data. “Empty” multilevel models were run for perpetration and victimization to calculate intraclass correlation coefficients (ICC) and design effects (Sommet & Morselli, 2017). For perpetration, the ICC was 0 and the design effect was 1. For victimization, the ICC was 0.01 and the design effect was 1.09. Since the ICCs were zero or negligible and the design effects were < 2, clustering in schools could be ignored and multilevel modeling was not needed (Muthén & Satorra, 1995; Sommet & Morselli, 2017).

### Descriptive Statistics and Gender Differences

Table 2 displays descriptive statistics for all study variables. Of the significant correlations between the sexualized media variables, most were positive, indicating that adolescents who consumed more of one type of sexualized media were also likely to consume more of other types of sexualized media. However, lower consumption of pornography was related to higher consumption of reality television. None of the sexualized media consumption variables were associated with adolescents’ endorsement of SDS norms. All correlations were small in size.

**Table 2:** Descriptive statistics for independent variables

Independent variables	1	2	3	4	5	Total sample	Girls	Boys	*t(df)*	Cohen’s *d*
						*M (SD)*	*M (SD)*	*M (SD)*		
1. Sexual music videos						2.13 (0.95)	2.22 (1.00)	2.00 (0.86)	− 1.77 (253.00)	*ns*
2. Pornography	.05^*^					2.30 (1.39)	1.69 (1.08)	3.16 (1.33)	9.40 (195.85)	1.24^***^
3. Sexually oriented reality TV	.19^**^	− .22^**^				1.77 (0.93)	2.01 (1.05)	1.43 (0.61)	− 5.52 (243.76)	− 0.65^***^
4. Sexy social media	.23^**^	.22^**^	− .05			3.88 (1.39)	4.00 (1.51)	3.72 (1.17)	− 1.66 (250.99)	*ns*
5. Total sexualized media exposure^a^	.67^**^	.48^**^	.42^**^	.64^**^		0.00 (0.55)	− 0.002 (0.58)	0.002 (0.51)	− 0.66 (253.00)	*ns*
6. Endorsement of SDS norms	.05	− .05	.05	.06	.05	0.47 (0.24)	0.47 (0.25)	0.46 (0.23)	− 0.56 (253.00)	*ns*

ns refers to non-significant.* t*-statistics and Cohen’s *d* refer to the difference between girls and boys on each study variable. Range of the media variables (numbers 1 − 4) = 1–7. Range of SDS norms =  − 1 to  + 1. ^a^Group mean for this variable is zero because it is a standardized composite of the different types of sexualized media consumption. ^*^p < .05. ^**^p < .01. ^***^p < .001

Independent *t-*tests revealed that boys watched pornography significantly more frequently than girls, whilst girls watched reality television significantly more frequently than boys. An independent *t-*test also indicated that the amount of sexual coercion victimization differed by gender, *t*(250.79) = -3.70, *p* < 0.001, *d* = − 0.45. Girls reported more victimization than boys. A chi-square test indicated that the proportion of sexual coercion perpetrators also differed by gender, χ^2^(1) = 4.90, *p* = 0.027, ϕ_c_ = 0.14, with 20.8% of boys reporting perpetration compared to 10.7% of girls. There were no gender differences on the other study variables.

### Sexual Coercion Perpetration

Table 3 displays the results of the hierarchical logistic regression model for sexual coercion perpetration predicted by gender, sexualized media consumption frequency, SDS norms, and the interactions between them.

**Table 3:** Hierarchical logistic regression results for sexual coercion perpetration

Variable	*B*	*SE B*	OR	95% *CI* for OR		*R*^*2*^*N*
				*LL*	*UL*	
Step 1						0.06
Gender^a^	− 0.83^*^	0.36	0.44	0.22	0.89	
Age	0.31	0.17	1.37	0.98	1.90	
Step 2						0.09
Gender	− 0.83^*^	0.36	0.44	0.21	0.89	
Age	0.31	0.18	1.36	0.96	1.92	
Sexualized media consumption	0.66^*^	0.33	1.93	1.02	3.67	
SDS norms	− 0.54	0.75	0.58	0.13	2.51	
Step 3						0.17
Gender	− 1.06^*^	0.43	0.35	0.15	0.80	
Age	0.34	0.18	1.41	0.98	2.01	
Sexualized media consumption	0.26	0.52	1.30	0.47	3.61	
SDS-stereotypes	2.48	1.27	11.91	0.99	143.72	
Gender * sexualized media consumption	0.64	0.72	1.89	0.46	7.72	
Gender * SDS norms	− 6.01^**^	1.81	0.01	< 0.01	0.09	
Sexualized media consumption * SDS norms	2.21	1.32	9.13	0.68	122.31	

SDS = sexual double standard; CI = confidence interval; *LL* = lower limit; *UL* = upper limit; *R*^*2*^*N* = Nagelkerke *R*^2^. ^a^Boys were the reference category. ^*^p < .05. ^**^p < .01

Including gender and the covariate age in Step 1 lead to a significant model, χ^2^(2) = 8.18, *p* = .017, which explained 6% of the variance in perpetration. Boys had greater odds of sexual coercion perpetration than girls.

In Step 2, sexualized media consumption and SDS norms were added. Although the model was significant, χ^2^(4) = 12.58, *p* = .014, including these variables led to only a marginally improved model fit, Δχ^2^(2) = 4.40, *p* = .111. The model explained 9% of the variance in perpetration. More sexualized media consumption was significantly associated with increased odds of sexual coercion perpetration. Endorsement of SDS norms was not associated with sexual coercion perpetration.

In Step 3, the three two-way interactions between gender, sexualized media consumption, and SDS norms were added. The likelihood ratio test was significant, Δχ^2^(3) = 13.11, *p* = .004, indicating that including these interactions significantly improved model fit. The model explained 17% of the variance in perpetration. Only the interaction between SDS norms and gender was significant, indicating that the association between SDS endorsement and sexual coercion perpetration was different for boys and girls. Decomposing the interaction showed that, for girls, stronger endorsement of SDS norms was associated with decreased odds of sexual coercion perpetration (*B* = − 2.95, *SE* = 1.10, Wald = 7.22, *p* = .007, OR = 0.52, 95% CI [0.01, 0.45]). However, for boys, stronger endorsement of SDS norms was associated with increased odds of sexual coercion perpetration, although this association was not significant in itself (*B* = 2.32, *SE* = 1.24, Wald = 3.51, *p* = .061, OR = 10.13, 95% CI [0.90, 114.07]).

Post-hoc sensitivity analyses to examine which types of sexualized media consumption contributed most to sexual coercion perpetration showed that greater consumption of pornography and reality television were both associated with increased odds of sexual coercion perpetration (porn: *B* = 0.50, *SE* = 0.16, Wald = 10.35, *p* = .001, OR = 1.65, 95% CI [1.22, 2.24]; reality tv: *B* = 0.43, *SE* = 0.21, Wald = 4.40, *p* = .036, OR = 1.54, 95% CI [1.03, 2.29]). Sexual coercion perpetration was not related to exposure to others’ sexy social media posts (*B* = − 0.09, *SE* = 0.18, Wald = 0.20, *p* = .655, OR = 0.91, 95% CI [0.91, 1.86]) or sexually explicit music videos (*B* = − 0.12, *SE* = 0.15, Wald = 0.67, *p* = .413, OR = 0.89, 95% CI [0.67, 1.18]).

### Sexual Coercion Victimization

Table 4 displays the results of the ordinal regression model for sexual coercion victimization predicted by gender, sexualized media consumption frequency, SDS norms, and the interactions between them.

**Table 4:** Ordinal regression results for sexual coercion victimization

Variable	*B*	*SE B*	Wald	OR	95% *CI* for OR	
					*LL*	*UL*
Model 1						
Gender^a^	0.99^***^	0.29	11.87	2.70	1.54	4.75
Education^b^						
Lower secondary level	− 0.85	1.00	0.72	0.43	0.05	3.86
Higher secondary level	− 1.03	1.01	1.04	0.36	0.04	3.25
Pre-university level	− 1.65	1.05	2.47	0.19	0.02	1.88
Model 2						
Gender^a^	1.03^***^	0.29	12.41	2.79	1.58	4.94
Education^b^						
Lower secondary level	− 1.22	1.03	1.41	0.30	0.03	2.69
Higher secondary level	− 1.55	1.06	2.14	0.21	0.02	2.03
Pre-university level	− 2.10	1.09	3.73	0.12	0.01	1.24
Sexualized media consumption	0.50^*^	0.25	4.07	1.65	1.02	2.70
SDS norms	0.35	0.55	0.40	1.41	0.48	4.19
Model 3						
Gender^a^	1.10^***^	0.30	13.39	2.99	1.66	5.37
Education^b^						
Lower secondary level	− 0.98	1.15	0.74	0.37	0.04	3.54
Higher secondary level	− 1.37	1.16	1.39	0.25	0.03	2.48
Pre-university level	− 1.86	1.20	2.42	0.16	0.02	1.63
Sexualized media consumption	0.67	0.49	1.84	1.96	0.74	5.15
SDS norms	− 0.40	1.07	0.14	0.67	0.08	5.51
Gender * sexualized media consumption	− 0.18	0.57	0.10	0.84	0.27	2.57
Gender * SDS norms	0.81	1.28	0.40	2.24	0.18	27.46
Sexualized media consumption * SDS norms	1.07	0.95	1.27	2.91	0.45	18.74

CI = confidence interval; *LL* = lower limit; *UL* = upper limit. ^a^Boys were the reference category. ^b^Grammar school represents the reference category. ^*^p < .05. ^***^p < .001

In Step 1, including gender and educational level as a covariate lead to a significant model, χ^2^(4) = 18.97, *p* < .001. Girls were more likely to experience sexual coercion victimization than boys.

In Step 2, sexualized media consumption and SDS norms were added. The model was significant, χ^2^(6) = 23.35, *p* < .001, but there was only marginal improvement in model fit, Δχ^2^(2) = 4.38, *p* = .112. More sexualized media consumption was significantly associated with increased odds of sexual coercion victimization.

In Step 3, the three two-way interactions between gender, sexualized media consumption, and SDS norms were added. The model was significant, χ^2^(9) = 25.47, *p* = .002, but there was no significant improvement in model fit, Δχ^2^(3) = 2.21, *p* = .548. None of the interactions were significant either. Therefore, the model in Step 2 without interactions was retained as the most parsimonious final model.

Post-hoc sensitivity analyses to examine which types of sexualized media consumption contributed most to sexual coercion victimization showed that greater consumption of others’ sexy social media posts was associated with increased odds of sexual coercion victimization (*B* = 0.32, *SE* = 0.11, Wald = 8.18, *p* = .004, 95% CI Wald [0.10, 0.53]). The other types of media were not significantly associated with sexual coercion victimization (porn: *B* = 0.01, *SE* = 0.12, Wald < 0.01, *p* = .004, 95% CI Wald [-0.23, 0.25]; reality tv: *B* = 0.01, *SE* = 0.15, Wald < 0.01, *p* = .964, 95% CI Wald [-0.29, 0.30]; music videos: *B* = 0.01, *SE* = 0.15, Wald < 0.01, *p* = .966, 95% CI Wald [-0.28, 0.29]).

## Discussion

This study examined interrelations between sexualized media consumption, SDS norms, gender, and sexually active adolescents’ sexual coercion perpetration and victimization. The first hypothesis was largely supported. Overall, greater consumption of sexualized media was associated with a greater likelihood of both sexual coercion perpetration and victimization in sexually active adolescents. More specifically, when adolescents viewed online pornography and sexually oriented reality television more frequently, they had increased odds of sexual coercion perpetration. When adolescents viewed others’ sexy social media posts more frequently, they had increased odds of sexual coercion victimization. Consumption frequency of sexually explicit music videos was not associated with either sexual coercion perpetration or victimization. However, the associations between sexualized media consumption and sexual coercion perpetration and victimization were not stronger for adolescents who more strongly endorsed SDS norms. In contrast to the second and third hypothesis, the associations between sexualized media consumption and sexual coercion perpetration and victimization were not different for boys and girls. In line with the fourth hypothesis, endorsement of SDS norms was associated with different odds of sexual coercion perpetration for girls and boys. When adolescents endorsed SDS norms, girls had lower odds of sexual coercion perpetration in comparison to boys. No support was found for the fifth hypothesis, as the association between endorsement of SDS norms and sexual coercion victimization was not different for boys and girls.

### Sexualized Media Consumption and Sexual Coercion

The findings that more exposure to several types of sexualized media are related to both sexual coercion perpetration and victimization are not surprising in light of previous content analyses of pornography, reality television, social media posts, and sexualized music videos (Birdsall & Keay, 2018; Bridges et al., 2010; Carrotte et al., 2017; Stevens Aubrey & Frisby, 2011). These media types often explicitly depict both sexually aggressive and dominant behaviors as well as permissive behavior and sexual objectification. Via processes such as modeling and self-objectification, adolescents might internalize the sexual behaviors they observe in the media into their own sexual scripts and repertoire (Bussey & Bandura, 1999; Karsay et al., 2018). This appears to make them susceptible to being a victim as well as a perpetrator of sexual coercion.

Interestingly, different types of sexualized media were associated with sexual coercion perpetration and victimization. This may indicate that there are different underlying mechanisms. The media types associated with perpetration, that is pornography and reality television, are those most likely to explicitly depict sexually coercive interactions between couples (Birdsall & Keay, 2018; Bridges et al., 2010; Morgan, 2021; Vera-Gray et al., 2021). Therefore, modeling may account for associations between sexualized media consumption and sexual coercion perpetration (Bussey & Bandura, 1999).

In contrast, the media type associated with sexual coercion victimization, that is others’ sexy social media posts, was unlikely to provide opportunities for modeling. The posts examined in the current study were still photographs, in which it would be difficult to depict sexually coercive interactions. Therefore, an alternative mechanism likely accounts for this association. Although exposure to sexualized media has been associated with self-objectification (Karsay et al., 2018), viewing others’ sexy social media posts was not associated with sexual objectification of girls in a previous Dutch study (van Oosten et al., 2015a, 2015b). Further research that directly tests self-objectification as a mechanism underlying the association between viewing sexy social media posts and sexual coercion victimization is therefore needed. There are also other possible explanations. Social media use is associated with a higher risk of depression (Liu et al., 2022), and depression is associated with later sexual assault victimization (Krahé & Berger, 2017). Therefore, depressive symptoms could also be examined as a potential mechanism.

The findings for specific media types being associated with sexual coercion perpetration were partially consistent with previous research. First, the association between viewing online pornography and sexual coercion perpetration was also found in male (Simons et al., 2012) and female (Kernsmith & Kernsmith, 2009) emerging adults, and adolescent boys (Stanley et al., 2018). Our finding shows that this association also pertains to adolescent girls. Second, the association between viewing sexually oriented reality television and sexual coercion perpetration is consistent with previous findings in male emerging adults (Seabrook et al., 2019). Our finding demonstrates that this association is also present in a younger age group, in girls as well as boys. No association was found between viewing sexually explicit music videos and sexual coercion perpetration, despite previously found associations with greater acceptance of both rape myths (Kistler & Lee, 2009) and violence against women (Stevens Aubrey et al., 2011). This may be because the current study focused on sexually coercive behavior, rather than attitudes as in previous research, which may not translate into actual perpetration of sexual coercion.

The findings for specific media types being associated with sexual coercion victimization were somewhat consistent with previous research. First, pornography consumption was not related to sexual coercion victimization. This finding was not entirely unexpected, as the current study included adolescent girls and boys and previous studies established an association in adult women only (de Heer et al., 2021; Simons et al., 2012). Maybe compared to adults, adolescents have not yet fully internalized the explicit sexual objectification present in pornography, which could increase victimization risk. Second, viewing sexually oriented reality television was not related to sexual coercion victimization, despite previous associations with sexualized television and film (Ybarra et al., 2014). Potentially, there are differences in the sexual content of fiction and reality programming that account for these different findings. In reality programming, sexual encounters are real and broadcasting rules only allow for more “implicit” showing of these encounters (e.g., under a blanket, blurred, often accompanied with actual audio). In contrast, sexual encounters in fiction programming are portrayed by actors, and no actual sexual contact is occurring. As the sex is only simulated, these sexual encounters can be broadcast despite their explicit nature. In line with Ybarra et al. (2014), viewing sexually explicit music videos was not related to sexual coercion victimization. This suggests that different forms of sexual victimization might have different predictors, as Ybarra et al. (2014) did find a significant association with rape victimization. This highlights the importance of studying sexual coercion as a separate element of sexual violence alongside rape or harassment. The finding that viewing others’ sexy social media posts was associated with increased odds of victimization is new, and adds to previous research on topics such as depression (e.g., Liu et al., 2022) and body image (e.g., Vuong et al., 2021), that have identified negative outcomes associated with social media use. However, it should be noted that adding sexualized media consumption to the model for sexual coercion victimization only resulted in a marginally improved model fit. Although the statistical test was influenced by the inclusion of the non-significant SDS endorsement variable and the non-significant other sexualized media types, the limited improvement in model fit suggests that viewing others’ sexy social media may only have small practical relevance to sexual coercion victimization, despite its statistical significance.

### Sexualized Media Consumption and Sexual Coercion: Moderation by Gender

Overall, our findings suggest that objectification cannot fully explain associations between sexualized media consumption and sexual coercion, as the associations were comparable for boys and girls. Even though the media objectifies women more than men (American Psychological Association, Task Force on the Sexualization of Girls, 2007), media consumption was not more strongly related to boys’ than girls’ sexual coercion perpetration. Similarly, even though greater objectification of women could lead to greater self-objectification among girls than boys (Fredrickson & Roberts, 1997), this did not lead to a stronger association between media consumption and girls’ than boys’ sexual coercion victimization. However, the lack of moderation by adolescent gender might also be due to an underestimation of the objectification of males in the media types examined in the current study. In social media “fitspiration” posts, sexualization was common for both men and women, although women were sexualized more often (Carrotte et al., 2017). Therefore, more research into the objectification of males in sexualized media is needed, as well as studies that directly test this mechanism in the relation between sexualized media consumption and sexual coercion.

### Endorsement of Sexual Double Standard and Sexual Coercion: Moderation by Gender

In addition to consumption of sexualized media, adolescents’ endorsement of SDS norms was also associated with sexual coercion perpetration, but in different ways for boys and girls. Not surprisingly, as traditional SDS norms dictate different sexual behaviors for men and women, that is male sexual dominance and female sexual passivity (Endendijk et al., 2020), girls who endorsed SDS norms were less likely to be perpetrators of sexual coercion. In comparison to these girls, boys who endorsed SDS norms were more likely to be perpetrators of sexual coercion. As such, both boys and girls seemed to have internalized societal SDS norms into their own sexual scripts and behavioral repertoire. Previous research did not find consistent gender differences in the associations between more general gender norms and attitudes and sexual coercion perpetration and victimization (Fernández-Fuertes et al., 2018; Krahé et al., 2015; Shen et al., 2012). More general gender norms and beliefs might be less meaningfully related to the sexual behavior of men and women than the endorsement of specific gender norms and scripts for sexuality.

That we did not find any associations between SDS norms and sexual coercion victimization could indicate that sexual scripts for passivity and submissiveness might be less gender-differentiated than sexual scripts for dominance and aggressiveness. Indeed, gender differences in reported sexual coercion victimization are often smaller or non-existent compared to gender differences in reported perpetration (Fernández-Fuertes et al., 2018; Krahé et al., 2015; Shen et al., 2012).

### Interaction Between Sexualized Media Consumption and Endorsement of Sexual Double Standard

Contrary to our expectations, there was no significant interaction between sexualized media consumption and endorsement of SDS norms for either sexual coercion perpetration or sexual coercion victimization. There are several potential explanations for why these interactions were not significant. Sexualized media often contains content that provides a model for the SDS as well as sexually coercive behaviors (Vandenbosch et al., 2015; Ward, 2003). In addition, media effects are theorized to be more likely when media content and attitudes are congruous (Valkenburg & Peter, 2013). However, because the SDS prescribes different behaviors for men/boys and women/girls, boys who endorse SDS norms expect themselves to be dominant and initiate (hetero)sexual activity, but girls who endorse SDS norms expect themselves to be passive and reactive. Consequently, the interaction between sexualized media consumption and endorsement of SDS norms could be different for males and females. Namely, greater consumption of sexualized media and stronger endorsement of SDS norms might lead to increases in sexual coercion perpetration in boys but not girls, and to increases in sexual coercion victimization in girls but not boys.

Second, sexualized media consumption and endorsement of SDS norms could be associated in a different way. It is possible that adolescents who more strongly endorse SDS norms choose to consume more sexualized media, as it portrays men and women in ways that align with their existing views.

An alternate explanation is that sexualized media consumption actually is associated with adolescents’ sexual coercion perpetration and victimization regardless of adolescents’ personal views regarding the SDS. This would be concerning, as it would mean a greater number of adolescents are vulnerable to negative outcomes associated with sexualized media consumption.

### Limitations and Future Directions

Our results should be interpreted in light of some limitations. First, the design of this study was correlational. Therefore, we cannot draw any conclusions about the direction of the associations we have identified. Whilst it is possible that viewing sexualized media increases the risk of sexual coercion perpetration and victimization, it is also possible that perpetrators and victims of sexual coercion choose to consume more of these types of media. Longitudinal data would provide more clarity as to the direction of effects. With longitudinal data it would also be possible to test possible mediation processes. For instance, sexualized media provides models for the SDS, which could be associated with more personal endorsement of SDS norms, which subsequently might be related to sexual coercion perpetration or victimization. Alternatively, adolescents who strongly endorse SDS norms might be more likely to watch sexualized media, which in turn is related to sexual coercion perpetration or victimization.

Second, due to the modest sample size and lower frequency of sexual coercion perpetration and victimization, we were not able to examine three-way interactions between the predictors of interest, i.e., sexualized media consumption, SDS norms, and gender. It would be interesting to examine the differential effects of the interaction between SDS norm endorsement and sexualized media consumption for boys’ and girls’ sexual coercion perpetration and victimization in a larger sample.

Third, our understanding of sexual coercion was limited by the measures used. Our survey contained only two questions on victimization, and one on perpetration. One of the victimization questions asked whether adolescents have felt “forced” to have sex. The perpetration question asked whether adolescents have “applied some pressure” in order to have sex. Adolescents may not interpret all coercive behaviors, such as use of lies or false promises, as “force” or “pressure”. This may have affected reporting of both perpetration and victimization, but also may partially explain the comparatively low number of participants who reported sexual coercion perpetration. When certain coercive tactics, such as emotional manipulation, are used, a victim may be aware that they feel forced, but the perpetrator may not be aware that they are exerting force. This may contribute to underreporting of sexual coercion perpetration by girls in particular, as women have been found to use more subtle verbal sexual coercion tactics than men and are also more likely to report unintentional coercion (Jeffrey & Senn, 2024). In addition, adolescents may not always honestly report perpetration even when they are aware of it. A larger number of questions, asking about specific coercive behaviors, might provide a more nuanced and multi-faceted assessment of sexual coercion perpetration and victimization.

Relatedly, the question “Have you ever felt forced to have sex when you didn’t really want to?” is open to interpretation in that it does not specify that the respondent should have “felt forced” by the person with whom they had sexual contact. It is possible, particularly given their young age, that participants felt forced to engage in sexual activity due to peer pressure or broader societal norms around sexual activity in adolescence. This could also have contributed to the substantially higher number of victims, both boys and girls, in our study compared to perpetrators.

Furthermore, it is unclear what proportion of sexual coercion experiences were completed sexual coercion and what proportion were attempted sexual coercion. Currently, one of the three questions is clearly about completed sexual coercion (“Have you ever had sex against your will while under the influence of alcohol or drugs?”), whilst for the other two questions participants could also report attempted sexual coercion. We recommend that the questions are revised so that they all either clearly assess completed sexual coercion or can all also assess attempted sexual coercion.

Finally, only sexually active adolescents were asked about sexual coercion perpetration and victimization during data collection. Sexual coercion victims with no consensual sexual experiences may not consider themselves sexually active and so may not have been included in this study. Future studies may wish to ask all participants about both attempted and completed sexual coercion perpetration and victimization.

### Conclusion

Viewing sexualized media and internalization of societal SDS norms may put sexually active adolescents at risk for sexual coercion experiences. The next step is to examine these associations in a longitudinal dataset, so that we can understand whether sexualized media consumption and endorsement of SDS norms precede sexual coercion experiences. Although the precise direction of these associations is yet unclear, there are practical implications of the current findings. It would be unrealistic to suggest that parents can prevent late adolescents from accessing several types of sexualized media. However, there may be value in discussing the consequences of exposure to sexualized media with adolescents, both at home and at school, as well as addressing endorsement of SDS norms that may increase risk of sexual coercion experiences. In that way, parents and schools can foster adolescents’ resilience to the normative influence of the media and the societal SDS. Producers and regulators of sexualized media may also want to more carefully consider the messages these media types may be sending to impressionable young people. It is important to highlight that more sexualized media types were associated with sexual coercion perpetration than with victimization, and that SDS norms were solely related to sexual coercion perpetration and not to victimization. This may be because adolescents’ have less control over their own victimization than their sexual coercion perpetration. Therefore, interventions aimed at reducing sexual coercion among adolescents may be more successful if they target media-related exposure and societal gender norms associated with perpetration, rather than victimization.

## Data Availability

The data are available upon request from the corresponding author.

## Appendix

See Table

**Table 5:** Correlations between study variables separately for boys and girls

Variable	1	2	3	4	5	6	7	8
1. Sexual music videos		.01	.29^**^	.13	.60^**^	.16	− .04	.09
2. Pornography	.21^**^		.01	.37^**^	.63^**^	.20^*^	.21^*^	− .15
3. Sexually oriented reality TV	.12	− .12		.08	.49^**^	.09	− .002	.10
4. Sexy social media	.27^**^	.29^**^	− .14		.67^**^	.19	.08	.10
5. Total sexualized media exposure^a^	.71^**^	.51^**^	.43^**^	.62^**^		.27^**^	.11	.05
6. Endorsement of SDS norms	− .02	− .22^**^	.03	− .01	− .08		.17	− .03
7. Sexual coercion perpetration	.02	.17^*^	.19^*^	− .06	.13	− .26^**^		.17
8. Sexual coercion victimization	− .004	.15	− .05	.18^*^	.11	.02	− .004	

Correlations below the diagonal are for girls, correlations above the diagonal are for boys. ^a^This variable is a composite of the different types of sexualized media consumption. ^*^*p* < .05. ^**^*p* < .01

^5^.
